# A Highly Accurate Pixel-Based FRAP Model Based on Spectral-Domain Numerical Methods

**DOI:** 10.1016/j.bpj.2019.02.023

**Published:** 2019-03-01

**Authors:** Magnus Röding, Leander Lacroix, Annika Krona, Tobias Gebäck, Niklas Lorén

**Affiliations:** 1 RISE Research Institutes of Sweden, Bioscience and Materials, Göteborg, Sweden; 2 Mathematical Sciences, Chalmers University of Technology, Göteborg, Sweden; 3 Department of Physics, Chalmers University of Technology, Göteborg, Sweden

## Abstract

We introduce a new, to our knowledge, numerical model based on spectral methods for analysis of fluorescence recovery after photobleaching data. The model covers pure diffusion and diffusion and binding (reaction-diffusion) with immobile binding sites, as well as arbitrary bleach region shapes. Fitting of the model is supported using both conventional recovery-curve-based estimation and pixel-based estimation, in which all individual pixels in the data are utilized. The model explicitly accounts for multiple bleach frames, diffusion (and binding) during bleaching, and bleaching during imaging. To our knowledge, no other fluorescence recovery after photobleaching framework incorporates all these model features and estimation methods. We thoroughly validate the model by comparison to stochastic simulations of particle dynamics and find it to be highly accurate. We perform simulation studies to compare recovery-curve-based estimation and pixel-based estimation in realistic settings and show that pixel-based estimation is the better method for parameter estimation as well as for distinguishing pure diffusion from diffusion and binding. We show that accounting for multiple bleach frames is important and that the effect of neglecting this is qualitatively different for the two estimation methods. We perform a simple experimental validation showing that pixel-based estimation provides better agreement with literature values than recovery-curve-based estimation and that accounting for multiple bleach frames improves the result. Further, the software developed in this work is freely available online.

## Introduction

Diffusive transport properties in complex, soft matter fluctuate spatially and temporally and depend strongly on the degree of heterogeneity, obstruction effects, structural dynamics, and interactions with a matrix, e.g., binding effects (1). Understanding complex diffusion phenomena is a recurring problem in several fields, and fluorescence recovery after photobleaching (FRAP) has emerged as a powerful technique to this end (2). Having been used for estimation of diffusion coefficients since the 1970s (3), FRAP has later been put to use on reaction-diffusion systems, joint estimation of diffusion coefficients, and (on and off) binding rate constants, i.e., association and disassociation rate constants. Different approaches to FRAP for quantifying diffusion and binding interactions have shed light on how proteins interact with binding sites within the cell and nucleus (4, 5, 6), the transcription factor mobility in the nucleus (7) and its interaction with chromatin (8, 9), interactions of membrane-associated proteins (10, 11, 12), and probe diffusion in *β*-lactoglobulin gels and solutions (13), just to mention a few.

In a typical FRAP experiment, a fluorescent species is irreversibly photobleached in either a circular or a rectangular bleach region. Unbleached particles will move into the bleach region at a rate governed by the mobility and interaction parameters. This leads to a recovery of fluorescence in the bleach region. Assuming that the bleaching does not significantly change the total amount of fluorescence in the sample and that no particles are immobile, the recovery will eventually be complete. A confocal laser scanning microscope (CLSM) is typically used to image the time evolution of the recovery, using the same laser for imaging and bleaching but with different intensity. Quantitative information is obtained by fitting a model for the fluorescence recovery to the experimental data. The physical/mathematical assumptions of the FRAP models as well as how the fitting is performed vary greatly between different approaches but boil down to representing the solution to a (reaction-)diffusion equation for the fluorescent species, analytically or numerically. We give a brief account of the literature for the factors that matter for the work below but refer the reader to the review in (2) for a more detailed account.

First, the prototypical FRAP approaches assuming pure diffusion are heavily used, but the generalizations incorporating interactions with binding sites facilitate the use of FRAP in more complex systems like cells and hydrogels. The governing model becomes a reaction-diffusion equation system with a “free” diffusion coefficient, binding and unbinding rate parameters, and possibly a “bound” diffusion coefficient, the latter depending on whether the binding sites are modeled as mobile (11, 12, 14) or immobile (6, 7, 8, 9).

Second, the bleach region theoretically has uniform intensity and is typically either a circle or a rectangle. For a uniform circular bleach region, the average intensity in the bleach region as a function of time after bleaching (i.e., the recovery curve) can be expressed in closed form using Bessel functions (15, 16). However, to obtain a closed-form expression for the full diffusion equation, i.e., the spatiotemporal evolution of the fluorescence intensity, a circular bleach region has to be approximated, e.g., by a Gaussian (17) or a nonparametric profile (18). For the rectangular case, the full solution to the diffusion equation is available in closed form (19, 20). Arbitrary bleach region shapes are in principle not a problem if numerical or Monte Carlo methods are used (21). Some approaches account for the effective, finite bleach resolution (because of a nonuniform laser beam) by convolving the bleach region by a Gaussian (16, 19).

Third, the duration of bleaching is non-negligible, and therefore diffusion (and binding) during bleaching affects the observed fluorescence recovery (22). The fact that very often multiple bleach frames are used (to increase the amount of bleaching and hence the contrast and signal/noise) and the fact that the laser moves in a raster scan pattern during bleaching (and imaging) both contribute to this effect. Diffusion during a single bleach frame can be accounted for implicitly to some extent by incorporating a bleach resolution parameter as a free fitting parameter (because both diffusion and the “smearing” of the bleach region by convolution with a Gaussian are mathematically equivalent) (19). It can also be accounted for explicitly by modifying the diffusion equation accordingly (11, 23). Multiple bleach frames have also been implemented (24). A very comprehensive model for the raster scan motion of the laser during bleaching (and imaging) is developed by (9). However, that approach results in computation times of several days for a single parameter set, and they are forced to rely on a database of precomputed numerical solutions instead of traditional estimation methods. Also (24) investigates the importance of modeling the scanning motion but concludes that diffusion during the typically multiple bleach frames is more important to model than diffusion during the scanning time of a single bleach frame. This is in line with a previous investigation of our own, which also suggested that the scanning motion during bleaching is to some extent counteracted by the scanning motion during imaging (25).

Fourth, bleaching during imaging can have substantial impact on the observed data and hence on estimated parameters and is frequently corrected for as part of the preprocessing by (exponential) rescaling and normalization. However, as has been pointed out, introducing corrections may lead to incorrect parameter estimates if the used FRAP model is not compatible with the correction, in particular if it is combined with a correction accounting for an immobile fraction (26). It is also crucial that corrections are performed in the appropriate order. Therefore, it is advantageous to explicitly incorporate bleaching during imaging into the model (27).

Fifth, estimation of parameters can be performed in a variety of ways. Most approaches use conventional recovery-curve-based estimation, meaning that the model is fitted to the time series of average fluorescence within the bleach region. However, models that provide the spatiotemporal evolution of concentration, i.e., the full solution to the (reaction-)diffusion equation, can be fitted using the intensity values of all individual pixels, utilizing spatiotemporal information instead of just temporal information (17, 18, 19, 20, 28, 29). Further, whereas ordinary least squares with the assumption of constant noise variance is very common, real experimental noise is more complex (16, 30), and some methods assume more realistically that the noise variance is proportional to the mean intensity (reflecting the underlying Poisson nature of the photon counts) (19) or a sum of the two (20).

In this work, we introduce a new, to our knowledge, numerical model based on spectral methods for analysis of FRAP data. Further, the model covers pure diffusion and diffusion and binding (reaction-diffusion) with immobile binding sites. Both circular and rectangular bleach regions are supported, with the option of supplying an arbitrary user-defined bleach region shape as well. Further, a bleach and imaging resolution parameter is included in the model. Fitting of the model is supported using both conventional recovery-curve-based estimation and pixel-based estimation, in which all individual pixels in the data are utilized. The model explicitly accounts for multiple bleach frames, diffusion (and binding) during bleaching, and bleaching during imaging. To our knowledge, no other FRAP framework incorporates all these model features and estimation methods. First, we thoroughly validate the model by comparison to stochastic simulations of particle dynamics and find it to be highly accurate. Second, we perform simulation studies to compare recovery-curve-based estimation and pixel-based estimation in realistic settings and show that pixel-based estimation is the better method for parameter estimation as well as for distinguishing pure diffusion from diffusion and binding. Third, we show that accounting for multiple bleach frames is important and that the effect of neglecting this is qualitatively different for the two estimation methods. Fourth, we study computational speed of the different models and estimation methods. Fifth, we perform a simple experimental validation using sodium fluorescein dissolved in water, showing that pixel-based estimation provides better agreement with literature values than recovery-curve-based estimation and that accounting for multiple bleach frames improves the result. The FRAP analysis code as well as the stochastic simulation code is freely available online at https://github.com/roding/frap_matlab.

## Methods

### Model

In a FRAP measurement, a fluorescent species is irreversibly photobleached in typically either a circular or a rectangular bleach region. Unbleached particles will move into the bleach region and bleached particles will move out at a rate governed by the mobility and interaction parameters. This leads to a recovery of fluorescence through the time evolution of the concentration of the (still) fluorescent species, *c*(*x*, *y*, *t*), from which quantitative information can be extracted.

Assuming that the bleach region is sufficiently extended in the axial dimension (by means of a small numerical aperture), net diffusion in the *z* direction, i.e., orthogonal to the focal plane, can be neglected, and only two-dimensional motion has to be considered. The concentration is initially *c*(*x*, *y*) = *c*_0_ everywhere. Immediately after the first bleach frame, the concentration is

(1) $$c\left(x,y\right)=\{\begin{array}{cc} c_{0}\alpha & ,\left(x,y\right)\in \Omega \\ c_{0} & ,\left(x,y\right)\notin \Omega \end{array},$$

where *α* is a bleaching parameter and *Ω* is the bleach region, centered in (*x*_*c*_, *y*_*c*_) and either circular with radius *r* or rectangular with dimensions *l*_*x*_ and *l*_*y*_. If more than one bleach frame is used, it becomes more complicated, and we cover that case in the numerical implementation below. For pure diffusion with a diffusion coefficient *D*, the evolution of the concentration *c*(*x*, *y*, *t*) is described by the standard diffusion equation

(2) $$\frac{\partial c}{\partial t}=D\nabla^{2}c.$$

Complementing the diffusion with an interaction between unbound particles (*U*) and binding sites (*S*) that together form bound complexes (*B*),

(3) $$U+S\overset{k_{\text{on}}^{⋆}}{\underset{k_{\text{off}}}{⇌}}B,$$

the observed concentration is the sum of the unbound and bound concentrations, *c* = *u* + *b*. Here, *k*_off_ is the off-binding rate and $k_{\text{on}}^{⋆}$ is the on-binding rate. Assuming a sufficiently high concentration of uniformly distributed immobile binding sites, the evolution of the concentration is described by two coupled first-order reaction-diffusion equations,

(4) $$\begin{array}{l} \frac{\partial u}{\partial t}=D\nabla^{2}u-k_{\text{on}}u+k_{\text{off}}b\\ \frac{\partial b}{\partial t}=k_{\text{on}}u-k_{\text{off}}b. \end{array}$$

Here, $k_{\text{on}}=k_{\text{on}}^{⋆}s_{\text{eq}}$, where *s*_eq_ is the equilibrium concentration of binding sites (we will use on-binding rate to denote *k*_on_ from now on). It follows that the average times of a fluorophore being unbound and bound are (7, 13, 31)

(5) $$\begin{array}{l} \mu_{u}=1/k_{\text{on}}.\\ \mu_{b}=1/k_{\text{off}} \end{array}$$

Also, it follows that the equilibrium concentrations of unbound and bound species are *u* = *π*_*u*_*c* and *b* = *π*_*b*_*c*, where

(6) $$\begin{array}{l} \pi_{u}=\frac{k_{\text{off}}}{k_{\text{on}}+k_{\text{off}}}.\\ \pi_{b}=\frac{k_{\text{on}}}{k_{\text{on}}+k_{\text{off}}} \end{array}$$

### Numerical solution

For the numerical solution, let the final simulated frame size be *N* × *N* (*N* = 256 throughout this work). Assume that the fluorescent species is contained in a two-dimensional box with periodic boundary conditions. To avoid periodicity artifacts, we perform the computations on an (*N* + 2*M*) × (*N* + 2*M*) grid, where *M* is the padding (*M* = 128 throughout this work). We solve Eqs. 2 and 4 using spectral methods. Time stepping is performed in the Fourier domain, and bleaching (including bleaching during imaging) is performed in the spatial domain. The numerical solution can be computed for arbitrary numbers of prebleach frames *n*_prebleach_, bleach frames *n*_bleach_, and postbleach frames *n*_postbleach_, with time lag *Δt* between consecutive frames. Bleaching is represented by an (*N* + 2*M*) × (*N* + 2*M*) matrix, which is 1 outside the bleach region, *α* inside, and some intermediate value for edge pixels. To accurately represent the edges of the bleach region, this matrix is supersampled, initialized at 15 times the final resolution, and downsampled through an averaging filter. Bleaching during imaging, governed by a parameter *β*, is represented by a similar matrix set to 1 in the padding area and *β* in the image frame region. Finite bleach and finite imaging resolutions are accounted for by convolving the bleach matrix with a Gaussian filter with SD *γ* (performed before the downsampling and hence in practice using a SD of 15 *γ*). Although we refer to *γ* as a bleach and imaging resolution parameter, it is rather an inverse resolution parameter. As described previously (16, 19), the bleach resolution and the imaging resolution are physically two different things and not equal. However, the Gaussian filtering of the bleach region, as it is implemented here, is mathematically equal to a Gaussian filtering of the entire image and accounts for the combined effect of a finite bleach and a finite imaging resolution under the assumption of linear photobleaching, i.e., that both bleaching and imaging are single-photon processes.

For the pure diffusion case in Eq. 2, the numerical solution proceeds as follows. The concentration *c*(*x*, *y*, *t*), with *t* corresponding to an arbitrary prebleach, bleach, or postbleach frame, is transformed to its spectral representation $\hat{c}\left(\xi ,\eta ,t\right)$ using fast Fourier transform. In the spectral domain, the single partial differential equation becomes (*N* + 2*M*)^2^ independent ordinary differential equations of the form

(7) $$\frac{\partial \hat{c}\left(\xi ,\eta ,t\right)}{\partial t}=-\left(\xi^{2}+\eta^{2}\right)D\hat{c}\left(\xi ,\eta ,t\right),$$

one for each grid point (*ξ*, *η*). The solution is explicitly available for any time (step), and because we want to make a jump *Δt* in time, it takes the form

(8) $$\hat{c}\left(\xi ,\eta ,t+\Delta t\right)=e^{-\left(\xi^{2}+\eta^{2}\right)D\Delta t}\hat{c}\left(\xi ,\eta ,t\right).$$

After time stepping, inverse fast Fourier transform is applied to obtain *c*(*x*, *y*, *t* + *Δt*). Bleaching (including bleaching during imaging) is applied by element-wise multiplication of the solution and either (or both) of the two bleach matrices (bleaching could, in principle, be applied in the Fourier domain but would involve a very costly convolution operation). To account for an immobile fraction of particles, a fraction *ϕ*_m_ ≤ 1 of mobile particles may be chosen. Diffusion propagation is performed only for the fraction *ϕ*_m_ ≤ 1, which is mobile. The immobile fraction is handled separately and entirely in the spatial domain (because the spectral domain is only used for diffusion propagation); the immobile particles are only bleached, and they neither diffuse nor bind.

For the diffusion and binding case in Eq. 4, the concentrations of unbound and bound species, *u*(*x*, *y*, *t*) and *b*(*x*, *y*, *t*), are transformed to their spectral counterparts $\hat{u}\left(\xi ,\eta ,t\right)$ and $\hat{b}\left(\xi ,\eta ,t\right)$. Consequently, the single partial differential equation system becomes (*N* + 2*M*)^2^ independent (vector-valued) ordinary differential equations of the form

(9) $$\frac{\partial }{\partial t}\left(\begin{array}{c} \hat{u}\left(\xi ,\eta ,t\right)\\ \hat{b}\left(\xi ,\eta ,t\right) \end{array}\right)=A\left(\begin{array}{c} \hat{u}\left(\xi ,\eta ,t\right)\\ \hat{b}\left(\xi ,\eta ,t\right) \end{array}\right),$$

one for each grid point (*ξ*, *η*), where

(10) $$A=\left(\begin{array}{cc} -\left(\xi^{2}+\eta^{2}\right)D-k_{\text{on}} & k_{\text{off}}\\ k_{\text{on}} & -k_{\text{off}} \end{array}\right).$$

The solution is once again explicitly available for any time (step), and because we want to make a jump *Δt* in time, it takes the form

(11) $$\left(\begin{array}{c} \hat{u}\left(\xi ,\eta ,t+\Delta t\right)\\ \hat{b}\left(\xi ,\eta ,t+\Delta t\right) \end{array}\right)=e^{A\Delta t}\left(\begin{array}{c} \hat{u}\left(\xi ,\eta ,t\right)\\ \hat{b}\left(\xi ,\eta ,t\right) \end{array}\right),$$

where

(12) $$e^{At}=\sum_{m=0}^{\infty }\frac{1}{m!}A^{m}t^{m}$$

is a matrix exponential. We make use of the eigendecomposition *A* = *QΛQ*^−1^ to obtain

(13) $$e^{At}=Q\left(\begin{array}{cc} e^{\Lambda \left(1,1\right)t} & 0\\ 0 & e^{\Lambda \left(2,2\right)t} \end{array}\right)Q^{-1},$$

for a diagonal eigenvalue matrix *Λ* (32). The elements of these matrices can be computed analytically as functions of *D*, *k*_on_, *k*_off_, *ξ*, and *η* (see Appendix A), providing for fast computations for the time stepping. Implementation of bleaching and an immobile fraction is performed identically to the pure diffusion case.

The numerical solver is implemented in MATLAB (The MathWorks, Natick, MA). The spectral method was found drastically more computationally efficient than finite difference methods, both explicit and implicit in time; further, the spectral method was found to work well and converge in the “native” resolution, i.e., the resolution of the final generated image, whereas our investigation into the other methods suggested that they would have to have been applied in higher spatial resolution and then downsampled, which would have been very costly. As will be shown below, the solutions are in excellent agreement with validation simulations.

### Stochastic simulation

For validation purposes, we also implement a stochastic model, where *n*_particles_ individual particles are simulated in a domain of size (*N* + 2*M*) × (*N* + 2*M*) with periodic boundary conditions directly corresponding to the computational grid in the numerical solver. The time evolution of a single particle is modeled as a discrete-time, continuous-space stochastic process with time step *Δt*_sim_ = *Δt*/*n* (*n* = 32 for diffusion and binding, and *n* = 1 for pure diffusion). A two-state (hidden) Markov model accounts for random switching between the unbound and bound states. The stationary distribution of the Markov chain, i.e., the marginal probabilities, is given by Eq. 6, according to which a random initial state is selected. The transition probabilities are dependent on the time step and equal to

(14) $$\begin{array}{l} p_{\text{u}\to \text{b}}=\Delta t_{\text{sim}}/\mu_{\text{u}}\\ p_{\text{b}\to \text{u}}=\Delta t_{\text{sim}}/\mu_{\text{b}}. \end{array}$$

The residence time distributions for the states are geometric with means given by Eq. 5 (in continuous time, the distributions would instead be exponential, with the same means). The initial particle position is uniformly distributed in the domain. In each time step, if the particle is at present unbound, it is displaced with a normal distributed increment with variance 2*DΔt*_sim_ in each direction; otherwise, it does not move. Also, a fraction 1 − *ϕ*_m_ of all particles remain fixed throughout the simulation. In every *n*th time step, if the particle is in the image frame (i.e., in [*M*, *N* + *M*] × [*M*, *N* + *M*]) and if it is not bleached, it is added to the corresponding pixel. The simulated FRAP image frame is formed as a histogram of particle positions. The algorithm is provided in a parallel implementation written in Julia 1.0.0 (www.julialang.org) (33). Most features of the numerical model are implemented in the stochastic model (one exception being finite bleach and imaging resolution for circular bleach regions).

### Noise models and parameter estimation

For a FRAP measurement with acquired data *c*_exp_(*x*, *y*, *t*) (*t* = 0 being the time of the first prebleach frame), estimation of parameters is performed in the following fashion. Because of the linearity of Eqs. 2 and 4 and because of the assumption that fluorescence is proportional to concentration, concentration and image intensity can be used interchangeably (although they are certainly not the same; the actual concentration is inaccessible in a FRAP measurement and not necessary for the modeling, either). Assume that the experimental noise is normally distributed and independent between pixels, with zero mean, and that the intensity variance *σ*^2^(*c*(*x*, *y*, *t*)) for a concentration *c*(*x*, *y*, *t*) is generally of the form

(15) $$\sigma^{2}\left(c\left(x,y,t\right)\right)=a+bc\left(x,y,t\right),$$

where *a* represents constant noise and *b* represents noise proportional to the mean intensity (reflecting the underlying Poisson nature of the photon counts) (16, 19, 20, 30). Let *θ* be the parameter vector, equal to *θ* = (*D*, *ϕ*_m_, *c*_0_, *α*, *β*, *γ*, *a*, *b*) (pure diffusion) or *θ* = (*D*, *k*_on_, *k*_off_, *ϕ*_m_, *c*_0_, *α*, *β*, *γ*, *a*, *b*) (diffusion and binding). For clarity, Table 1 lists all the parameters used in the models.

**Table 1:** Listing of All Parameters Used in the Models

Parameters used in both models	
*D*	diffusion coefficient
*ϕ*_m_	mobile fraction
*c*_0_	initial concentration/intensity
*α*	bleach parameter
*β*	imaging bleach parameter
*γ*	bleach and imaging resolution
*A*	constant noise parameter
*b*	proportional noise parameter

Parameters used only for diffusion and binding	

*k*_on_	on-binding rate
*k*_off_	off-binding rate

Typically, not all parameters have to be estimated from the data. For example, sometimes it is known that *ϕ*_m_ = 1 because the presence of an immobile fraction would be unphysical or that *β* is sufficiently close to 1 to set *β* = 1 because there are no signs of bleaching during imaging. Also, the resolution *γ* can be estimated from independent calibration data as described by Smisdom et al. (16), in which the bleach resolution contribution is estimated from a line-FRAP measurement using a reference solution with known diffusion coefficient (34), and the imaging resolution contribution is estimated from imaging of fixed subresolution beads (however, Smisdom et al. (16) recommend estimation directly from the FRAP data using a series of bleach region sizes; this option is not implemented in the current version of our code). Further, if constant noise variance is assumed, *b* = 0. If *b* > 0, *a* and *b* would preferably be estimated from independent calibration data from a homogeneous fluorescent solution, with varying laser intensities, otherwise using settings identical to those for the FRAP experiment (20). In consequence, the dimensionality of *θ* would often be reduced from the above “worst cases.”

For pixel-based estimation, the likelihood function (i.e., the joint probability distribution of all pixel intensities)

(16) $$L\left(\theta \right)=\prod_{x,y,t}\frac{1}{{\left(2\pi \sigma^{2}\left(c\left(x,y,t\right)\right)\right)}^{1/2}}\times \text{exp}\left(-\frac{{\left(c_{\text{exp}}\left(x,y,t\right)-c\left(x,y,t\right)\right)}^{2}}{2\sigma^{2}\left(c\left(x,y,t\right)\right)}\right)$$

or rather, the log-likelihood,

(17) $$l\left(\theta \right)=\text{log}L\left(\theta \right)=-\frac{1}{2}\sum_{x,y,t}\text{log}\left(2\pi \sigma^{2}\left(c\left(x,y,t\right)\right)\right)-\frac{1}{2}\sum_{x,y,t}\frac{{\left(c_{\text{exp}}\left(x,y,t\right)-c\left(x,y,t\right)\right)}^{2}}{\sigma^{2}\left(c\left(x,y,t\right)\right)},$$

is used (Eq. 17) (we suppress that *c*(*x*, *y*, *t*) depends on *θ* in the notation). Here, the product and the sums are over all the pixels in all (prebleach and postbleach) frames. Maximization of *l*(*θ*) yields maximal likelihood parameter estimates. For *b* = 0, i.e., when the noise variance is assumed to be constant, the log-likelihood function simplifies to the negative sum of squared residuals, and maximal likelihood estimation simplifies to ordinary least squares.

For recovery-curve-based estimation, we first compute the experimental recovery curve *F*_exp_(*t*) by

(18) $$F_{\text{exp}}\left(t\right)=\sum_{x,y}w\left(x,y\right)c_{\text{exp}}\left(x,y,t\right).$$

Here, *w*(*x*, *y*) is a normalized indicator function (matrix) of the bleach region such that Eq. 18 produces the average intensity inside the bleach region. The model recovery curve *F*(*t*) is computed similarly. The assumption of zero-mean normal distributed noise at the pixel level implies zero-mean normal distributed noise also at the recovery curve level. The variance can be computed by

(19) $$\sigma^{2}\left(F\left(t\right)\right)=\sum_{x,y}w{\left(x,y\right)}^{2}\left(a+bc\left(x,y,t\right)\right),$$

leading to the log-likelihood

(20) $$l\left(\theta \right)=-\frac{1}{2}\sum_{t}\log\left(2\pi \sigma^{2}\left(F\left(t\right)\right)\right)-\frac{1}{2}\sum_{x}\frac{{\left(F_{\text{exp}}\left(t\right)-F\left(t\right)\right)}^{2}}{\sigma^{2}\left(F\left(t\right)\right)}$$

(we suppress that *F*(*t*) depends on *θ* in the notation). Once again, for *b* = 0 (i.e., when the noise variance is assumed to be constant), the log-likelihood function simplifies to the negative sum of squared residuals, and maximal likelihood estimation coincides with ordinary least squares estimation. Even for recovery-curve-based estimation, *a* and *b* describe the pixel intensity noise.

In this context, it is worth to also point out the common problem of laser intensity fluctuations during the measurement. The fluctuations cannot be modeled but rather have to be empirically corrected for as a preprocessing step by normalizing the intensity in each frame with respect to a reference region (a set of pixels) placed sufficiently far away from the bleach region (19). This will also compensate for imaging bleach, and hence *β* should be set to 1 in the fitting.

The estimation is implemented in MATLAB (The MathWorks). If *b* is set to zero, lsqnonlin is used; otherwise, fmincon is used. If prebleach data is provided as input, which is optional, it is used for the estimation; prebleach data does not provide information about *D*, *k*_on_, *k*_off_, *α*, or *γ* but does provide information about *ϕ*_m_, *c*_0_, and *β* (and *a* and *b*).

### Limitations of the framework

We briefly summarize assumptions made in our FRAP framework to clarify its limitations: we assume 1) that the bleach region has a uniform intensity, 2) that binding sites are immobile (and also that they are uniformly distributed), 3) that the raster scanning motion of the CLSM during both bleaching and imaging has negligible impact on the acquired data (in terms of introducing asymmetry), 4) that the bleach region is sufficiently extended in the axial dimension by means of a small numerical aperture (so that only two-dimensional motion has to be considered), 5) that photobleaching is linear, 6) that finite bleaching and imaging resolution can be accurately summarized in a single resolution parameter, and 7) that noise can be accurately modeled as independent Gaussian noise with a variance generally having both constant and intensity-proportional terms. Otherwise, our FRAP framework is highly generic and should be able to accurately model any FRAP measurement.

## Results and Discussion

In all simulation studies, unless stated otherwise, we use a resolution *N* = 256 (256 × 256 pixel image frames) and pixel size *Δx* = 0.75 *μ*m (providing a field of view of 192 × 192 *μ*m), time lag *Δt* = 0.2 s between consecutive frames, and an initial concentration *c*_0_ = 1 (arbitrary units), whereas other parameters vary. Diffusion coefficients will vary from 5 × 10^−12^ to 5 × 10^−10^ m^2^/s; this is the range we typically observe in experiments. Rate constants *k*_on_ and *k*_off_ will vary from 0.05 to 5 s^−1^; the rationale is that the timescales covered by the data ranges from *Δt* = 0.2 s to 100*Δt* = 20 s (50*Δt* = 10 s in some cases), and the reciprocals of these values, i.e., 5 and 0.05 s^−1^, give an indication as to the range in which rate constants should be estimable using these data. These ranges for *D*, *k*_on_, and *k*_off_ are further selected because they cover the typical range of values we have observed in soft materials, e.g., in gels (13); other systems, e.g., cells, will result in different typical parameter ranges but also in different experimental settings to begin with, such as pixel size and bleach region size.

### Model validation

**Figure 1:**
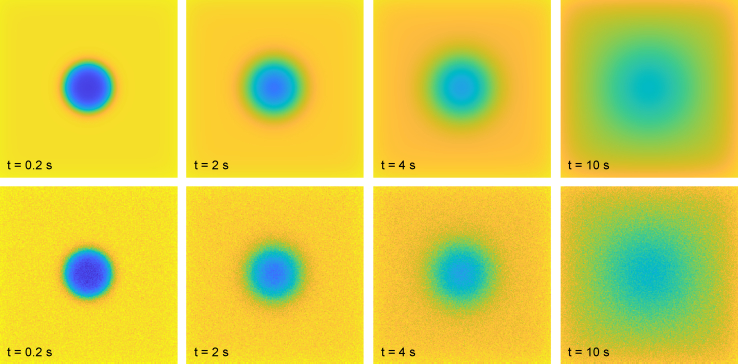
Comparison of numerical solution (*top*) and stochastic solution (*bottom*) using *n*_particles_ = 10^9^ particles. The mean-squared residual difference between the pixel-wise values of the numerical and stochastic solutions is approximately 2.5 × 10^−4^. The times indicated are relative to the time of the last bleach frame. To see this figure in color, go online.

**Figure 2:**
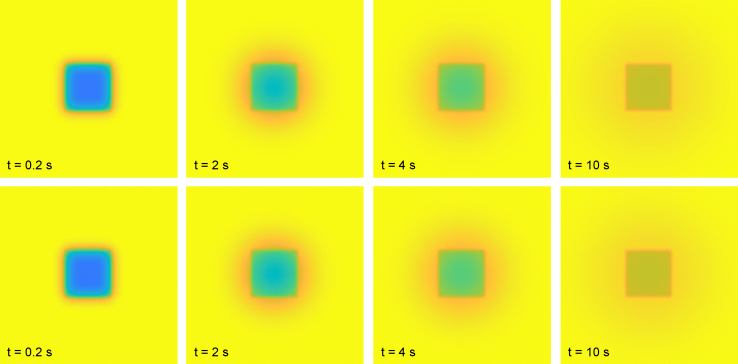
Comparison of numerical solution (*top*) and stochastic solution (*bottom*) using *n*_particles_ = 10^11^ particles. The mean-squared residual difference between the pixel-wise values of the numerical and stochastic solutions is approximately 2.5 × 10^−6^. The times indicated are relative to the time of the bleach frame. To see this figure in color, go online.

We validate the numerical solver by performing a comprehensive comparison between numerical solutions to Eqs. 2 and 4 and stochastic solutions for many different parameter values. We simulate with *n*_prebleach_ = 10 and *n*_postbleach_ = 50, using *n*_bleach_ = 4 with bleach parameter *α* = 0.9, resolution parameter *γ* = 0, and a circular bleach region with *r* = 25 *μ*m. Simulations are performed for *D* values {5 × 10^−12^, 10^−11^, 5 × 10^−11^, 10^−10^, 5 × 10^−10^} m^2^/s, *ϕ*_m_ values {0.8, 1}, and *β* values {0.998, 1}, both for pure diffusion and for diffusion and binding with *k*_on_ and *k*_off_ in {0.05, 0.1, 0.5, 1, 5} s^−1^ (but only for the combinations in which the difference between *k*_on_ and *k*_off_ is at most a factor of 10; this is due to the fact that if the difference is larger, the fraction of mobile particles will be close to either 0 or 1, and hence those cases are less interesting). Hence, the numerical and stochastic solutions are studied for 400 different parameter values. The stochastic simulations are performed using *n*_particles_ = 10^9^ particles, which takes on average 8 min using a dual Intel Xeon E5-2699 v4 running 88 threads. Because the resulting image frames from the stochastic simulations are histograms of particle counts, they are normalized by multiplying with (*N* + *M*)^2^*c*_0_/*n*_particles_ to be directly comparable with the numerical solutions. The mean-squared residual difference between the pixel-wise values of the numerical and stochastic solutions are approximately 2.5 × 10^−4^ for all simulations. One example is shown in Fig. 1 for diffusion and binding with *D* = 5 × 10^−11^ m^2^/s, *k*_on_ = 0.05 s^−1^, *k*_off_ = 0.5 s^−1^, *ϕ*_m_ = 1, and *β* = 0.998. It is worth noting that the apparent square-like concentration profile 10 s after bleaching in this case is due to imaging bleach and not due to artificial periodicity/boundary effects. Indeed, we compute the numerical solution for the same parameters but with a padding of 2048 pixels instead of 128: the largest pixel-wise difference in absolute value between the two solutions is 1.6 × 10^−10^, proving that periodicity/boundary effects are negligible. In addition, we investigate different bleach region sizes, rectangular bleach regions, finite bleach and imaging resolutions (*γ* > 0), and other numbers of bleach frames, but not as systematically as above. One example is shown in Fig. 2 for diffusion with *D* = 10^−10^ m^2^/s, *ϕ*_m_ = 0.8, and *β* = 1, using a rectangular (*square*) bleach region with *l* = 50 *μ*m, *γ* = 2 pixels, and *n*_bleach_ = 1 with bleach parameter *α* = 0.7. Here, *n*_particles_ = 10^11^ particles, which takes ∼5 h; for this large number of particles, the stochastic simulation is virtually indistinguishable from the numerical solution.

**Figure 3:**
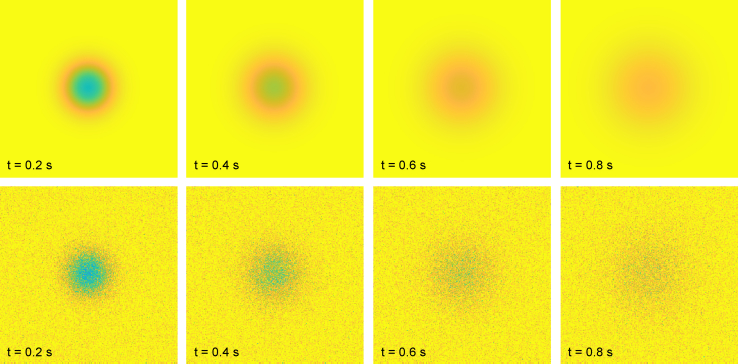
Comparison of numerical solution (*top*) and stochastic solution (*bottom*) using *n*_particles_ = 10^9^ particles. The mean-squared residual difference between the pixel-wise values of the numerical and stochastic solutions is approximately 2.5 × 10^−4^. The times indicated are relative to the time of the bleach frame. The field of view is 25.6 × 25.6 *μ*m, which is different from the other validation cases to study the dynamics around this smaller rectangular bleach region with *l* = 3 *μ*m more accurately. To see this figure in color, go online.

Another example is found in Fig. 3 and is more akin to how FRAP is performed in cells, with diffusion coefficient *D* = 10^−11^ m^2^/s, using a rectangular bleach region with *l* = 3 *μ*m, a modified pixel size *Δx* = 0.1 *μ*m (providing a field of view of 25.6 × 25.6 *μ*m to be able to study the dynamics around this smaller bleach region more accurately), a bleach and imaging resolution parameter *γ* = 10 pixels (equal to 1 *μ*m), and *n*_bleach_ = 1 with bleach parameter *α* = 0.7. Only the first four postbleach frames are shown in this case because the bleach region effectively vanishes very quickly (note also that although the bleach region is rectangular, it obtains a near-circular profile already in the first postbleach frame). The difference between the numerical and stochastic solutions is as small as for the other validation cases. The validation confirms that there is no problem with the numerical scheme (such as numerical diffusion) and that the bleach region is represented with high accuracy. That the pixel-wise difference is small implies that the difference in terms of recovery curves is also small.

### Comparison of estimation methods

We perform a comparison of pixel-based estimation and recovery-curve-based estimation by generating large numbers of simulated data sets for each of a number of parameters, adding noise, and estimating the parameters using both estimation methods. With zero noise, both methods will return the exact, true parameter values, i.e., both methods will perform equally. For nonzero noise, they will behave differently, however.

**Figure 4:**
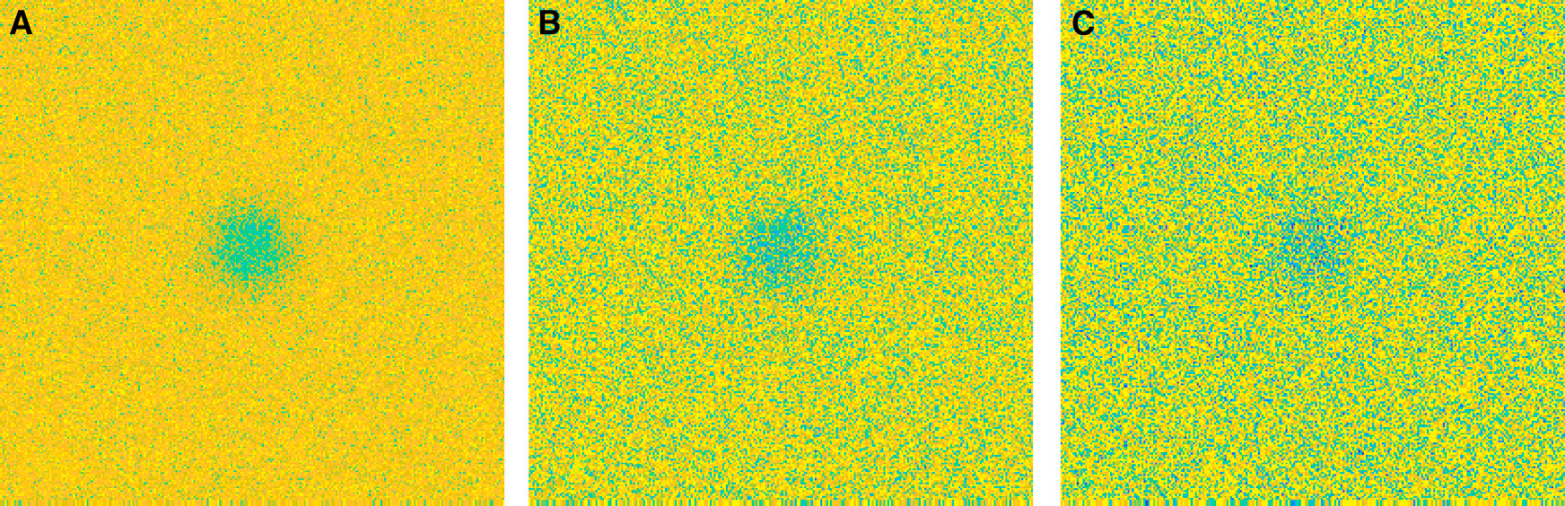
Examples of the first postbleach frame for noise levels (*A*) *a* = 0.01, (*B*) *a* = 0.05, and (*C*) *a* = 0.10. To see this figure in color, go online.

**Figure 5:**
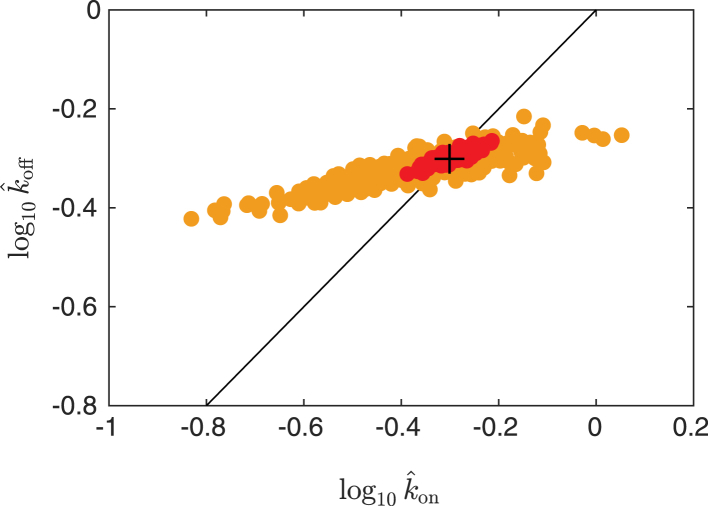
Distribution of ${\hat{k}}_{\text{on}}$ and ${\hat{k}}_{\text{off}}$ for both recovery-curve-based estimation (*yellow*) and pixel-based estimation (*red*) for *D* = 5 × 10^−10^ m^2^/s, *k*_on_ = 0.5 s^−1^, *k*_off_ = 0.5 s^−1^, and *a* = 0.01. The true parameters are indicated (*black plus sign*) as well as the line *k*_on_ = *k*_off_ (*black*). To see this figure in color, go online.

We generate data with *n*_prebleach_ = 10 and *n*_postbleach_ = 100, using *n*_bleach_ = 4 with bleach parameter *α* = 0.7^0.25^ (to make the total bleaching roughly equivalent to 30%, which is a realistic value and well below 50%, which is a rough upper limit for ensuring linear bleaching) and a circular bleach region with *r* = 15 *μ*m. Data is generated for *D* values {5 × 10^−12^, 10^−11^, 5 × 10^−11^, 10^−10^, 5 × 10^−10^} m^2^/s, both for pure diffusion and for diffusion and binding with *k*_on_ and *k*_off_ in {0.05, 0.1, 0.5, 1, 5} s^−1^ (but only for the combinations in which the difference between *k*_on_ and *k*_off_ is at most a factor of 10). This is performed for mobile fraction *ϕ*_m_ = 1, imaging bleach parameter *β* = 1, and bleach and imaging resolution *γ* = 0. We use a constant noise variance, letting *b* = 0 and using the *a*-values {0.01, 0.05, 0.1}; see Fig. 4. In total, this means we investigate 300 different parameters. For each parameter, *n* = 250 simulations, and subsequent estimations are performed. We are only concerned with the estimates of *D*, *k*_on_, and *k*_off_; for *D*, the recovery-curve-based and the pixel-based estimates are denoted ${\hat{D}}^{\left(\text{rc}\right)}$ and ${\hat{D}}^{\left(\text{px}\right)}$, respectively, and equivalently for *k*_on_ and *k*_off_. To quantify the error made in estimation, we compute the means and SDs of the estimates, denoted $M\left({\hat{D}}^{\left(\text{rc}\right)}\right)$ and $S\left({\hat{D}}^{\left(\text{rc}\right)}\right)$, for the recovery-based estimates of *D*, and equivalently for the others, and we also compute the mean-squared error defined by

(21) $$\text{MSE}\left({\hat{D}}^{\left(\text{rc}\right)}\right)=\langle {\left({\hat{D}}^{\left(\text{rc}\right)}-D\right)}^{2}\rangle ,$$

where *D* is the true value, and equivalently for all other cases. The mean-squared error is the sum of the variance and the squared bias of the estimate and hence is a summary of both precision (variance) and accuracy (bias). We need the mean-squared errors for comparison of the recovery-curve-based and the pixel-based estimates, for which we compute the so-called relative efficiency (of the pixel-based versus the recovery-curve-based estimate), which is the ratio of the mean-squared errors,

(22) $$E_{R}\left(D\right)=\text{MSE}\left({\hat{D}}^{\left(\text{px}\right)}\right)/\text{MSE}\left({\hat{D}}^{\left(\text{rc}\right)}\right),$$

and equivalently for *k*_on_ and *k*_off_. If *E*_*R*_ < 1, the pixel-based estimate performs better, and vice versa. For pure diffusion, results are shown in Table 2. The “worst-case” result (as in worst for pixel-based estimation, relative to recovery-based) is also indicated; hence, it can be seen that the pixel-based estimation performs better than the recovery-curve-based estimation in all cases. For diffusion and binding, an exhaustive presentation of all the results would be painstaking, so we present a small subset of the results for 10 cases, divided into Tables 3, 4, and 5, for *D*, *k*_on_, and *k*_off_, respectively. The “worst-case” results (as in worst for pixel-based estimation, relative to recovery-based, and for the entire simulation study, not just for the part shown herein) are also indicated for each of the three parameters; hence, it can be seen that the pixel-based estimation performs better than the recovery-curve-based estimation in all cases. It is not surprising that pixel-based estimation provides better results given that pixel-based estimation uses the full spatiotemporal data available. It has been pointed out that recovery-curve-based parameter estimation in diffusion and binding models suffers from instability simply because of the fact that a whole range of parameters yields approximately the same recovery curve (11). It appears that this problem can be substantially reduced by using pixel-based estimation instead. To further illustrate this, consider Fig. 5, in which the distributions of ${\hat{k}}_{\text{on}}$ and ${\hat{k}}_{\text{off}}$ are shown for both estimation methods for *D* = 5 × 10^−10^ m^2^/s, *k*_on_ = 0.5 s^−1^, *k*_off_ = 0.5 s^−1^, and *a* = 0.01: pixel-based estimation yields a substantially more narrow distribution of estimates than recovery-curve-based estimation. Further, the pixel-based estimates lie closer to the line *k*_on_ = *k*_off_; because *k*_on_ and *k*_off_ are equal in this example case, the unbound and bound proportions *π*_*u*_ and *π*_*b*_ are correctly estimated for parameters along that line. Indeed, pixel-based estimation yields substantially better estimates for the proportions as well (data not shown).

**Table 2:** Comparison of Recovery-Curve-Based and Pixel-Based Estimation for Pure Diffusion and for Some Different Parameters

*D* (m^2^/s)	*a*	$M\left({\hat{D}}^{\left(\text{rc}\right)}\right)$	$S\left({\hat{D}}^{\left(\text{rc}\right)}\right)$	$M\left({\hat{D}}^{\left(\text{px}\right)}\right)$	$S\left({\hat{D}}^{\left(\text{px}\right)}\right)$	*E*_*R*_(*D*)
5 × 10^−12^	0.01	5.12 × 10^−12^	1.92 × 10^−13^	5.01 × 10^−12^	2.58 × 10^−14^	0.015
5 × 10^−12^	0.05	5.30 × 10^−12^	4.51 × 10^−13^	5.03 × 10^−12^	5.58 × 10^−14^	0.013
5 × 10^−12^	0.10	5.40 × 10^−12^	6.33 × 10^−13^	5.04 × 10^−12^	8.03 × 10^−14^	0.014
1 × 10^−11^	0.01	1.01 × 10^−11^	2.57 × 10^−13^	1.00 × 10^−11^	5.28 × 10^−14^	0.037
1 × 10^−11^	0.05	1.03 × 10^−11^	5.46 × 10^−13^	1.01 × 10^−11^	1.15 × 10^−13^	0.041
1 × 10^−11^	0.10	1.05 × 10^−11^	8.33 × 10^−13^	1.01 × 10^−11^	1.65 × 10^−13^	0.035
5 × 10^−11^	0.01	5.05 × 10^−11^	1.63 × 10^−12^	5.01 × 10^−11^	3.94 × 10^−13^	0.058^a^
5 × 10^−11^	0.05	5.13 × 10^−11^	3.93 × 10^−12^	5.02 × 10^−11^	8.39 × 10^−13^	0.044
5 × 10^−11^	0.10	5.15 × 10^−11^	5.69 × 10^−12^	5.04 × 10^−11^	1.28 × 10^−12^	0.051
1 × 10^−10^	0.01	1.01 × 10^−10^	5.37 × 10^−12^	1.00 × 10^−10^	1.05 × 10^−12^	0.038
1 × 10^−10^	0.05	1.03 × 10^−10^	1.28 × 10^−11^	1.01 × 10^−10^	2.29 × 10^−12^	0.032
1 × 10^−10^	0.10	1.05 × 10^−10^	1.82 × 10^−11^	1.01 × 10^−10^	3.28 × 10^−12^	0.031
5 × 10^−10^	0.01	5.24 × 10^−10^	1.31 × 10^−10^	5.02 × 10^−10^	1.14 × 10^−11^	0.007
5 × 10^−10^	0.05	5.74 × 10^−10^	2.92 × 10^−10^	5.04 × 10^−10^	2.50 × 10^−11^	0.007
5 × 10^−10^	0.10	6.77 × 10^−10^	4.55 × 10^−10^	5.06 × 10^−10^	3.36 × 10^−11^	0.005

a This number is the “worst case.”

**Table 3:** Comparison of Recovery-Curve-Based and Pixel-Based Estimation for Diffusion and Binding and for Some Different Parameters, Showing Results for Estimation of *D*

*D* (m^2^/s)	*k*_on_ (s^−1^)	*k*_off_ (s^−1^)	*a*	$M\left({\hat{D}}^{\left(\text{rc}\right)}\right)$	$S\left({\hat{D}}^{\left(\text{rc}\right)}\right)$	$M\left({\hat{D}}^{\left(\text{px}\right)}\right)$	$S\left({\hat{D}}^{\left(\text{px}\right)}\right)$	*E*_*R*_(*D*)
5 × 10^−12^	0.05	0.05	0.01	5.12 × 10^−12^	8.30 × 10^−13^	4.99 × 10^−12^	9.18 × 10^−14^	0.012
5 × 10^−12^	1.00	5.00	0.01	5.05 × 10^−12^	4.12 × 10^−13^	5.01 × 10^−12^	1.82 × 10^−13^	0.192
1 × 10^−11^	5.00	1.00	0.10	1.48 × 10^−11^	1.37 × 10^−11^	1.01 × 10^−11^	8.85 × 10^−13^	0.004
5 × 10^−11^	0.10	0.05	0.01	5.82 × 10^−11^	2.82 × 10^−11^	5.01 × 10^−11^	8.81 × 10^−13^	0.001
5 × 10^−11^	0.05	0.10	0.05	7.03 × 10^−11^	4.38 × 10^−11^	5.00 × 10^−11^	1.59 × 10^−12^	0.001
1 × 10^−10^	0.05	0.10	0.05	1.49 × 10^−10^	1.29 × 10^−10^	1.00 × 10^−10^	3.23 × 10^−12^	0.001
1 × 10^−10^	0.50	5.00	0.10	1.72 × 10^−10^	1.38 × 10^−10^	1.01 × 10^−10^	8.71 × 10^−12^	0.003
5 × 10^−10^	0.50	0.05	0.01	6.21 × 10^−10^	3.37 × 10^−10^	5.04 × 10^−10^	2.76 × 10^−11^	0.006
5 × 10^−10^	5.00	0.50	0.01	5.26 × 10^−10^	5.61 × 10^−11^	5.08 × 10^−10^	4.02 × 10^−11^	0.438^a^
1 × 10^−10^	0.50	5.00	0.01	1.06 × 10^−10^	1.33 × 10^−11^	1.00 × 10^−10^	3.84 × 10^−12^	0.070

a This number is the “worst case.”

**Table 4:** Comparison of Recovery-Curve-Based and Pixel-Based Estimation for Diffusion and Binding and for Some Different Parameters, Showing Results for Estimation of *k*_on_

*D* (m^2^/s)	*k*_on_ (s^−1^)	*k*_off_ (s^−1^)	*a*	$M\left({\hat{k}}_{\text{on}}^{\left(\text{rc}\right)}\right)$	$S\left({\hat{k}}_{\text{on}}^{\left(\text{rc}\right)}\right)$	$M\left({\hat{k}}_{\text{on}}^{\left(\text{px}\right)}\right)$	$S\left({\hat{k}}_{\text{on}}^{\left(\text{px}\right)}\right)$	*E*_*R*_(*k*_on_)
5 × 10^−12^	0.05	0.05	0.01	0.051	0.011	0.050	0.002	0.042
5 × 10^−12^	1.00	5.00	0.01	0.921	0.208	1.008	0.118	0.280
1 × 10^−11^	5.00	1.00	0.10	4.580	1.293	5.088	0.466	0.122
5 × 10^−11^	0.10	0.05	0.01	0.100	0.009	0.101	0.003	0.085
5 × 10^−11^	0.05	0.10	0.05	0.059	0.017	0.051	0.005	0.058
1 × 10^−10^	0.05	0.10	0.05	0.053	0.011	0.051	0.004	0.134
1 × 10^−10^	0.50	5.00	0.10	0.982	1.136	0.574	0.331	0.076
5 × 10^−10^	0.50	0.05	0.01	0.524	0.166	0.507	0.039	0.057
5 × 10^−10^	5.00	0.50	0.01	4.870	0.490	5.098	0.480	0.935^a^
1 × 10^−10^	0.50	5.00	0.01	0.588	0.326	0.528	0.171	0.263

a This number is the “worst case.”

**Table 5:** Comparison of Recovery–Curve-Based and Pixel-Based Estimation for Diffusion and Binding and for Some Different Parameters, Showing Results for Estimation of *k*_off_

*D* (m^2^/s)	*k*_on_ (s^−1^)	*k*_off_ (s^−1^)	*a*	$M\left({\hat{k}}_{\text{off}}^{\left(\text{rc}\right)}\right)$	$S\left({\hat{k}}_{\text{off}}^{\left(\text{rc}\right)}\right)$	$M\left({\hat{k}}_{\text{off}}^{\left(\text{px}\right)}\right)$	$S\left({\hat{k}}_{\text{off}}^{\left(\text{px}\right)}\right)$	*E*_*R*_(*k*_off_)
5 × 10^−12^	0.05	0.05	0.01	0.052	0.011	0.050	0.001	0.020
5 × 10^−12^	1.00	5.00	0.01	5.639	2.572	5.387	2.445	0.872^a^
1 × 10^−11^	5.00	1.00	0.10	2.203	3.437	1.022	0.103	0.001
5 × 10^−11^	0.10	0.05	0.01	0.051	0.005	0.050	0.001	0.048
5 × 10^−11^	0.05	0.10	0.05	0.109	0.018	0.103	0.006	0.112
1 × 10^−10^	0.05	0.10	0.05	0.108	0.014	0.103	0.005	0.139
1 × 10^−10^	0.50	5.00	0.10	7.497	11.109	5.885	2.987	0.075
5 × 10^−10^	0.50	0.05	0.01	0.050	0.004	0.050	0.001	0.103
5 × 10^−10^	5.00	0.50	0.01	0.487	0.029	0.502	0.006	0.040
1 × 10^−10^	0.50	5.00	0.01	6.269	5.194	5.249	0.920	0.032

a This number is the “worst case.”

It should be noted that pixel-based estimation will not give a better fit than recovery-curve-based estimation in terms of the recovery curve; on the contrary, it will be slightly worse, although the visual difference is typically not obvious.

That pixel-based estimation performs better than recovery-curve-based estimation for parameter estimation leads to the question of whether it performs better for distinguishing between pure diffusion and diffusion and binding as well. We investigate this using the Akaike information criterion (AIC) (35) for model selection in a small part of the study performed above, namely for *D* = 5 × 10^−11^ m^2^/s and binding with *k*_on_ and *k*_off_ in the same combinations as above. Briefly, the model that minimizes

(23) $$\text{AIC}=2n_{\text{param}}+n_{\text{data}}\text{logRSS}$$

is considered the “best” model (in terms of AIC). Here, *n*_param_ is the number of parameters in the models (three for pure diffusion and five for diffusion and binding in this setting), *n*_data_ is the number of fitted data points (110 for recovery-curve-based and 110 × 256^2^ for pixel-based), and RSS is the residual sum of squares or the sum of squared differences between the data and the model. In all the investigated cases, the probability of selecting the correct model (i.e., diffusion and binding) is higher for pixel-based estimation. We provide a couple of examples in which the difference is substantial: for *k*_on_ = 0.05 s^−1^, *k*_off_ = 0.5 s^−1^, and *a* = 0.05, the probability is 0.09 for recovery-curve-based and 0.64 for pixel-based estimation, and for *k*_on_ = 5 s^−1^, *k*_off_ = 5 s^−1^, and *a* = 0.05, the probability is 0.02 for recovery-curve-based and 0.73 for pixel-based estimation. This small investigation provides yet another convincing argument for using pixel-based estimation.

### Bias when neglecting the number of bleach frames

**Figure 6:**
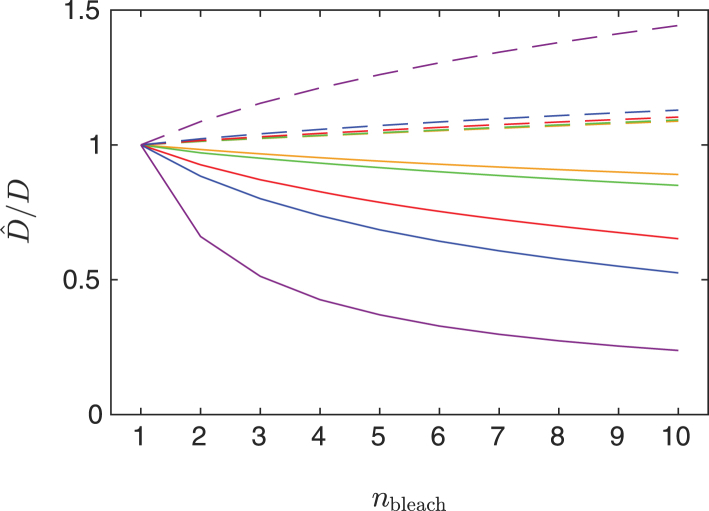
Relative estimated values of *D*, $\hat{D}/D$, for recovery-curve-based estimation (*solid lines*) and pixel-based estimation (*dashed lines*) assuming that *n*_bleach_ = 1 and as a function of the true value of *n*_bleach_, for *D*-values 5 × 10^−12^ m^2^/s (*yellow*), 10^−11^ m^2^/s (*green*), 5 × 10^−11^ m^2^/s (*red*), 10^−10^ m^2^/s (*blue*), and 5 × 10^−10^ m^2^/s (*purple*). To see this figure in color, go online.

Because the duration of bleaching is non-negligible, diffusion (and binding) during bleaching will impact parameter estimation if it is not appropriately accounted for. Frequently, multiple bleach frames are used, but very few models explicitly account for it. Here, we study the bias in parameter estimates as a function of the true number of bleach frames when the fitted model only assumes a single bleach frame. We generate data with *n*_prebleach_ = 10 and *n*_postbleach_ = 100, using *n*_bleach_ = 1–10 with bleach parameter *α* = 0.7(1/*n*_bleach_) (to make the total bleaching roughly equivalent to 30%), and a circular bleach region with *r* = 15 *μ*m. Data is generated for *D* values {5 × 10^−12^, 10^−11^, 5 × 10^−11^, 10^−10^, 5 × 10^−10^} m^2^/s (pure diffusion), mobile fraction *ϕ*_m_ = 1, imaging bleach parameter *β* = 1, and bleach and imaging resolution *γ* = 0. We do not add noise to the data because the aim is to study only the bias and not the variance of the estimates; hence, *a* = *b* = 0. The model is fitted with the incorrect assumption that *n*_bleach_ = 1. We are only concerned with the estimates of *D*, i.e., ${\hat{D}}^{\left(\text{rc}\right)}$ and ${\hat{D}}^{\left(\text{px}\right)}$, and compute the ratio $\hat{D}/D$; see Fig. 6. Note that if *n*_bleach_ were correctly specified in the fitted model, the exact parameter values would be obtained for any value of *n*_bleach_. Obviously, the error increases as *n*_bleach_ increases; it also increases as *D* increases because the particles diffuse increasingly much during the bleaching phase. Further, pixel-based estimation yields smaller errors than recovery-curve-based estimation. Interestingly, recovery-curve-based estimation produces too small values of *D*, whereas pixel-based estimation produces too large values.

### Computational speed

**Figure 7:**
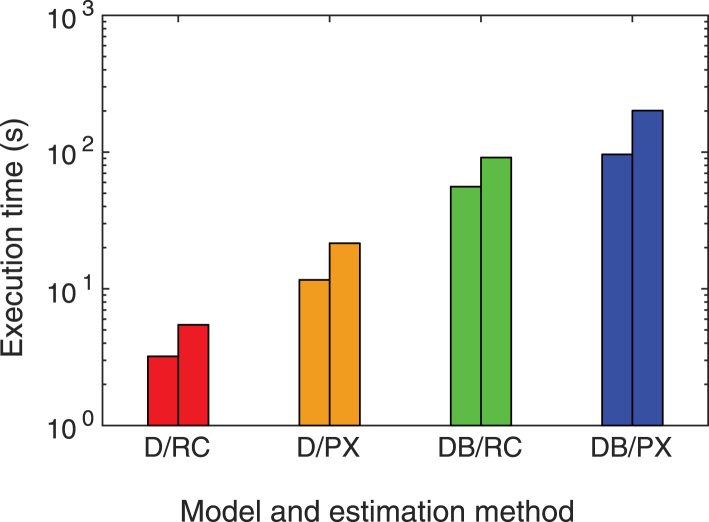
Mean execution time for pure diffusion and recovery-curve-based estimation (D/RC), pure diffusion and pixel-based estimation (D/PX), diffusion and binding and recovery-curve-based estimation (DB/RC), and diffusion and binding and pixel-based estimation (DB/PX), for *n*_postbleach_ = 50 (*left*) and *n*_postbleach_ = 100 (*right*). To see this figure in color, go online.

We study the execution time of performing one fit to data in different conditions. We generate data with *n*_prebleach_ = 10 and *n*_postbleach_ = 100, using *n*_bleach_ = 4 with bleach parameter *α* = 0.7^0.25^ and a circular bleach region with *r* = 15 *μ*m. Simulations are performed for random values of the parameters, with *D* values chosen log-uniformly distributed in the range [5 × 10^−12^, 5 × 10^−10^] m^2^/s, and for diffusion and binding, with *k*_on_ and *k*_off_ in the range [0.05, 5] s^−1^. This is performed for mobile fraction *ϕ*_m_ = 1, imaging bleach parameter *β* = 1, and bleach and imaging resolution *γ* = 0. We use a constant noise variance, using *b* = 0 and random values of *a* in the range [0, 0.1]. The investigation is performed for recovery-curve-based estimation and pixel-based estimation and for *n*_postbleach_ = 50 and *n*_postbleach_ = 100. In each case, *n* = 250 simulations and subsequent estimations are performed. The results are shown in Fig. 7. Not surprisingly, the diffusion and binding model is the more computationally demanding, as is the pixel-based estimation, and uses a larger number of postbleach frames for estimation. It is worth mentioning in this context that because of the use of spectral methods, the execution speed is similar to the analytical pixel-based approach of Deschout et al. (19), although our approach is much more generic and performed numerically, and faster than the numerical pixel-based approach of Jonasson et al. (18) (comparisons not shown).

### Arbitrary bleach region

**Figure 8:**
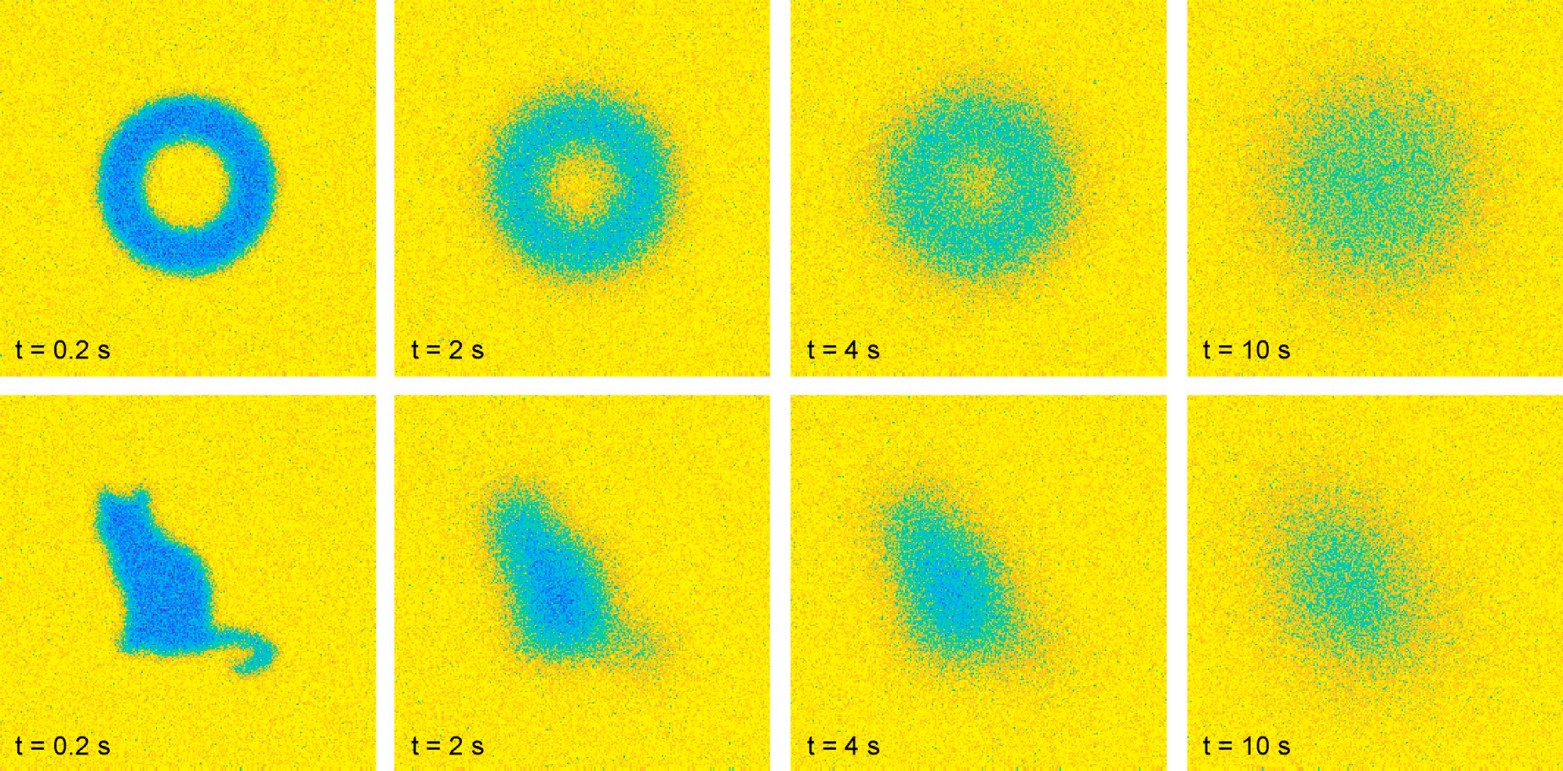
The software also supports arbitrary bleach regions, such as a torus (*top*) or a cat (*bottom*). The times indicated are relative to the time of the bleach frame. To see this figure in color, go online.

We briefly illustrate the ability of the FRAP code to, on top of circular and rectangular bleach regions, support completely arbitrary bleach region shapes. To illustrate this, we generate simulated data *n*_bleach_ = 1 and *α* = 0.7, *D* = 2.5 × 10^−12^ m^2^/s, *ϕ*_m_ = 1, *β* = 1, *γ* = 0, and *a* = 0.0025. The bleach region shape is in this case provided (by the user) as an indicator matrix of size (*N* + 2*M*) × (*N* + 2*M*), which is 1 inside the bleach region and 0 outside, and as can be seen in Fig. 8, the bleach region can then take any shape, such as a torus or a cat.

### Experimental validation

**Figure 9:**
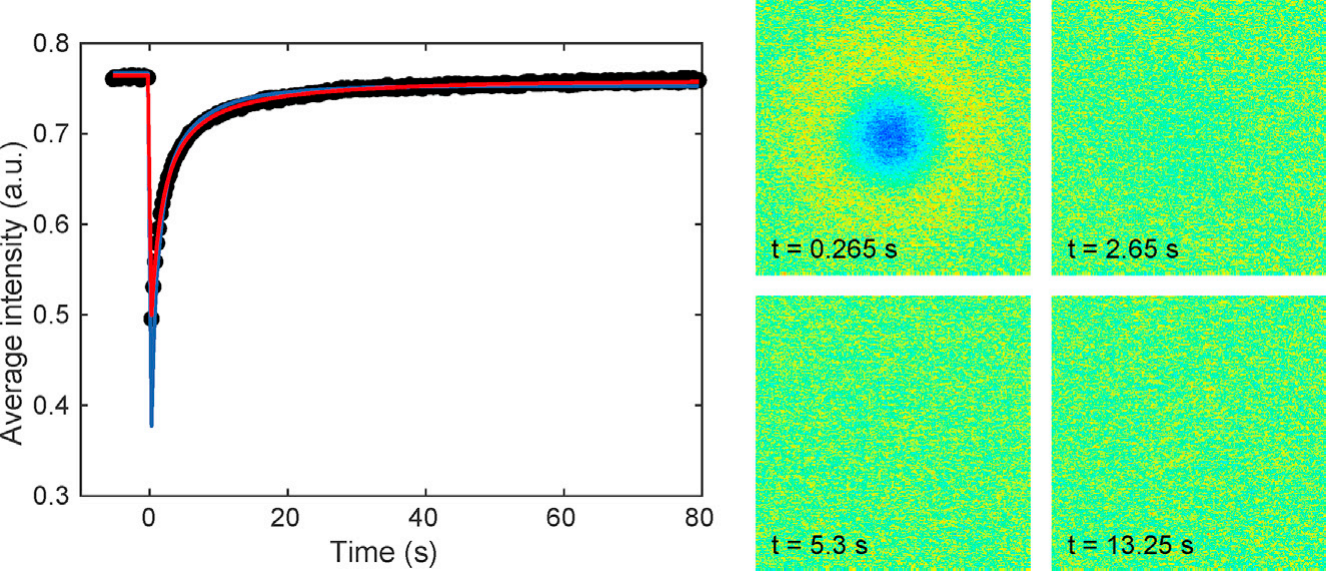
Estimation results for one of the experiments, showing recovery curves (*left*) for recovery-curve-based (*red*) and pixel-based (*blue*) estimation, as well as some residual postbleach images for pixel-based estimation (*right*). To see this figure in color, go online.

For experimental validation of the method, we perform FRAP measurements on sodium fluorescein salt (Sigma-Aldrich, St. Louis, MO) dissolved in water (the concentration of sodium fluorescein is 100 ppm, or 0.01 w/w %). Two different samples are prepared by placing 7 *μ*L of the solution in SecureSeal spacers (Grace Bio Labs, Bend, OR), and six measurements are performed in each sample on different locations, yielding 12 replicates in total. Measurements are performed at ambient conditions on a Leica SP5 CLSM (Leica, Heidelberg, Germany) using a Leica HCX APO 20×/0.50 water immersion lens at zoom 4 and pinhole size 6 Airy units. A 488 nm laser at 10% power and 1% acousto-optic tunable filter and a photomultiplier tube with gain 436 V are used for acquisition in the 500–650 nm range. The acquired image size is 256 × 256 pixels with field of view 193.75 *μ*m (pixel size is 0.76 *μ*m), obtaining 20 prebleach and 300 postbleach frames, and using four bleach frames with a circular bleach region with radius *r* = 25 *μ*m. The scan rate is 1000 Hz, yielding *Δt* = 0.265 s. Background subtraction is performed by subtracting a pixel-wise Gaussian filtered (*σ* = 5 pixels) prebleach average frame from all pre- and postbleach frames and adding the average prebleach intensity back again. There is little to no indication of bleaching during imaging or laser intensity fluctuations, and hence, no corrections are introduced. The estimated diffusion coefficients are (m ± SD) 1.47 ± 0.17 × 10^−10^ m^2^/s using recovery-curve-based estimation and 4.06 ± 0.12 × 10^−10^ m^2^/s using pixel-based estimation. In Fig. 9, results from one of the measurements are shown. We note that the recovery-curve-based estimation yields a better fit to the recovery curve, which is expected. On the other hand, pixel-based estimation yields a better fit to the images, i.e., to the actual FRAP data (the residual images from the recovery-curve-based estimation are not shown). Although the recovery curve can be replicated more or less perfectly by the model, there is a lack of fit observed in the residual images just after bleaching. We stress that this lack of fit, possibly due to imperfections in the bleach region definition, usually remains unobserved because typically only recovery-curve-based estimation is performed. Therefore, comparison in this regard to other models is not possible. To give an idea of the magnitude of the error, the SD of this first postbleach residual image is 0.05 (for *c*_0_ ≈ 0.77). Further, the result from the pixel-based estimation is in much better agreement with literature values from non-FRAP methods, e.g., 3.9 ± 0.4 × 10^−10^ m^2^/s using NMR at 27°C (36) and 4 × 10^−10^ m^2^/s using two-photon flash photolysis at 20°C (37) (it is also interesting, although strictly not for validation, that the value 4.2 × 10^−10^ m^2^/s has been obtained using molecular dynamics simulations at 300 K (38)). To illustrate the usefulness of explicitly modeling the true number of bleach frames, we attempt estimation under the incorrect assumption of only one bleach frame and obtain 1.15 ± 0.10 × 10^−10^ m^2^/s using recovery-curve-based estimation and 3.59 ± 0.10 × 10^−10^ m^2^/s using pixel-based estimation, which in both cases is further away from the reference values than the results for the true number of bleach frames. We stress that although the difference between the two estimation methods illustrates that pixel-based estimation can indeed be superior to recovery-curve-based estimation, it is not indicative of the difference between the estimation methods for all experimental parameters and/or samples; the difference may be substantially smaller in other cases.

## Conclusions

We have implemented a new, to our knowledge, numerical model based on spectral methods for analysis of FRAP data. The model is highly generic and covers both pure diffusion and diffusion and binding (reaction-diffusion) with immobile binding sites, arbitrary bleach region shapes, and conventional recovery-curve-based as well as pixel-based estimation and accounts for multiple bleach frames, diffusion (and binding) during bleaching, and bleaching during imaging. To our knowledge, no other FRAP framework incorporates all these model features and estimation methods. The model is thoroughly validated by comparison to stochastic simulations of particle dynamics and is found to be highly accurate. Additionally, simulation studies indicated that pixel-based estimation is superior to recovery-curve-based estimation for parameter estimation as well as for distinguishing pure diffusion from diffusion and binding. Further, we demonstrate the importance of accounting for multiple bleach frames and that the effect of neglecting this is qualitatively different for the two estimation methods. Also, we perform a simple experimental validation showing that pixel-based estimation provides better agreement with literature values than recovery-curve-based estimation and that accounting for multiple bleach frames improves the result. Finally, the developed software is made freely available online, which facilitates widespread use of this new, to our knowledge, FRAP model. Interesting further work would be to characterize the ranges of parameter values in which reliable estimates can be provided for different experimental settings.

## Author Contributions

M.R. developed and implemented methods, performed simulations, and wrote the manuscript. L.L. developed and implemented methods together with M.R. A.K. performed sample preparation and the experimental FRAP measurements. T.G. contributed to and supervised method development. N.L. contributed to manuscript writing and simulation study design.

## Appendix A: Eigendecomposition

The matrix *A* in Eqs. 12 and 13 can be written *A* = *QΛQ*^−1^, where

(24) $$\begin{array}{r} Q\left(1,1\right)=-(k_{\text{on}}-k_{\text{off}}+(D^{2}{\left(\xi^{2}+\eta^{2}\right)}^{2}+2Dk_{\text{on}}\left(\xi^{2}+\eta^{2}\right)-\ldots \\ {2Dk_{\text{off}}\left(\xi^{2}+\eta^{2}\right)+k_{\text{on}}^{2}+2k_{\text{on}}k_{\text{off}}+k_{\text{off}}^{2})}^{1/2}+D\left(\xi^{2}+\eta^{2}\right))/\left(2k_{\mathrm{on}}\right)\\ Q\left(1,2\right)=-(k_{\text{on}}-k_{\text{off}}-(D^{2}{\left(\xi^{2}+\eta^{2}\right)}^{2}+2Dk_{\text{on}}\left(\xi^{2}+\eta^{2}\right)-\ldots ,\\ {2Dk_{\text{off}}\left(\xi^{2}+\eta^{2}\right)+k_{\text{on}}^{2}+2k_{\text{on}}k_{\text{off}}+k_{\text{off}}^{2})}^{1/2}+D\left(\xi^{2}+\eta^{2}\right))/\left(2k_{\mathrm{on}}\right)\\ Q\left(2,1\right)=1\\ Q\left(2,2\right)=1 \end{array}$$

$$\text{Λ}\left(1,1\right)=-(k_{\text{on}}+k_{\text{off}}+(D^{2}{\left(\xi^{2}+\eta^{2}\right)}^{2}+2Dk_{\text{on}}\left(\xi^{2}+\eta^{2}\right)-\ldots$$

(25) $$\begin{array}{r} {2Dk_{\text{off}}\left(\xi^{2}+\eta^{2}\right)+k_{\text{on}}^{2}+2k_{\text{on}}k_{\text{off}}+k_{\text{off}}^{2})}^{1/2}+D\left(\xi^{2}+\eta^{2}\right))/2\\ \text{Λ}\left(1,2\right)=0\\ \text{Λ}\left(2,1\right)=0\\ \text{Λ}\left(2,2\right)=-(k_{\text{on}}+k_{\text{off}}-(D^{2}{\left(\xi^{2}+\eta^{2}\right)}^{2}+2Dk_{\text{on}}\left(\xi^{2}+\eta^{2}\right)-\ldots \\ {2Dk_{\text{off}}\left(\xi^{2}+\eta^{2}\right)+k_{\text{on}}^{2}+2k_{\text{on}}k_{\text{off}}+k_{\text{off}}^{2})}^{1/2}+D\left(\xi^{2}+\eta^{2}\right))/2 \end{array}$$

and

(26) $$\begin{array}{r} Q^{-1}\left(1,1\right)=-k_{\text{on}}/(2k_{\text{on}}k_{\text{off}}+k_{\text{on}}^{2}+k_{\text{off}}^{2}+D^{2}{\left(\xi^{2}+\eta^{2}\right)}^{2}+\ldots \\ {2Dk_{\text{on}}\left(\xi^{2}+\eta^{2}\right)-2Dk_{\text{off}}\left(\xi^{2}+\eta^{2}\right))}^{1/2}\\ Q^{-1}\left(1,2\right)=-(k_{\text{on}}-k_{\text{off}}-(D^{2}{\left(\xi^{2}+\eta^{2}\right)}^{2}+2Dk_{\text{on}}\left(\xi^{2}+\eta^{2}\right)-\ldots \\ {2Dk_{\text{off}}\left(\xi^{2}+\eta^{2}\right)+k_{\text{on}}^{2}+2k_{\text{on}}k_{\text{off}}+k_{\text{off}}^{2})}^{1/2}+D\left(\xi^{2}+\eta^{2}\right))/\ldots \\ \left(2{\left(2k_{\text{on}}k_{\text{off}}+k_{\text{on}}^{2}+k_{\text{off}}^{2}+D^{2}{\left(\xi^{2}+\eta^{2}\right)}^{2}+2Dk_{\text{on}}\left(\xi^{2}+\eta^{2}\right)-2Dk_{\text{off}}\left(\xi^{2}+\eta^{2}\right)\right)}^{1/2}\right)\\ Q^{-1}\left(2,1\right)=k_{\text{on}}/(2k_{\text{on}}k_{\text{off}}+k_{\text{on}}^{2}+k_{\text{off}}^{2}+D^{2}{\left(\xi^{2}+\eta^{2}\right)}^{2}+\ldots \\ {2Dk_{\text{on}}\left(\xi^{2}+\eta^{2}\right)-2Dk_{\text{off}}\left(\xi^{2}+\eta^{2}\right))}^{1/2}\\ Q^{-1}\left(2,2\right)=(k_{\text{on}}-k_{\text{off}}+(D^{2}{\left(\xi^{2}+\eta^{2}\right)}^{2}+2Dk_{\text{on}}\left(\xi^{2}+\eta^{2}\right)-\ldots \\ {2Dk_{\text{off}}\left(\xi^{2}+\eta^{2}\right)+k_{\text{on}}^{2}+2k_{\text{on}}k_{\text{off}}+k_{\text{off}}^{2})}^{1/2}+D\left(\xi^{2}+\eta^{2}\right))/\ldots \\ \left(2{\left(2k_{\text{on}}k_{\text{off}}+k_{\text{on}}^{2}+k_{\text{off}}^{2}+D^{2}{\left(\xi^{2}+\eta^{2}\right)}^{2}+2Dk_{\text{on}}\left(\xi^{2}+\eta^{2}\right)-2Dk_{\text{off}}\left(\xi^{2}+\eta^{2}\right)\right)}^{1/2}\right). \end{array}$$
